# Assessing the impact of law enforcement to reduce over-the-counter (OTC) sales of antibiotics in low- and middle-income countries; a systematic literature review

**DOI:** 10.1186/s12913-019-4359-8

**Published:** 2019-07-31

**Authors:** Tom G. Jacobs, Jane Robertson, Hendrika A. van den Ham, Kotoji Iwamoto, Hanne Bak Pedersen, Aukje K. Mantel-Teeuwisse

**Affiliations:** 1 WHO Collaborating Centre for Pharmaceutical Policy and Regulation, Division of Pharmacoepidemiology & Clinical Pharmacology, Utrecht Institute for Pharmaceutical Sciences (UIPS), Utrecht University, David de Wied building, Universiteitsweg 99, 3584 CG Utrecht, The Netherlands; 2 World Health Organization (WHO) Regional Office for Europe, UN City, Marmorvej 51, 2100 Copenhagen, Denmark; 3 University of Newcastle Calvary Mater Hospital, Edith St & Platt St, Waratah NSW, Newcastle, 2298 Australia

**Keywords:** Law enforcement, Antibiotics, Over-the-counter, Prescription-only, Policy change, Impact measurement

## Abstract

**Background:**

Many low- and middle-income countries (LMIC) are moving towards enforcing prescription-only access to antibiotics. This systematic literature review aims to assess the interventions used to enforce existing legislation prohibiting over-the-counter (OTC) sales of antibiotics in LMICs, their impact and examine the methods chosen for impact measurement including their strengths and weaknesses.

**Methods:**

Both PubMed and Embase were systematically searched for studies reporting on impact measurement in moving towards prescription only access to antibiotics in LMICs. The PRISMA methodological review framework was used to ensure systematic data collection and analysis of literature. Narrative data synthesis was used due to heterogeneity of study designs.

**Results:**

In total, 15 studies were included that assessed policy impact in 10 different countries. Strategies employed to enforce regulations prohibiting OTC sales of systemic antibiotics included retention of prescriptions for antibiotics by pharmacies, government inspections, engaging pharmacists in the design of interventions, media campaigns for the general public and educational activities for health care workers. A variety of outcomes was used to assess the policy impact; changes in antimicrobial resistance rates, changes in levels of antibiotic use, changes in trends of antibiotic use, changes in OTC supply of antibiotics, and changes in reported practices and knowledge of pharmacists, medicine sellers and the general public. Differences in methodological approaches and outcome assessment made it difficult to compare the effectiveness of law enforcement activities. Most effective appeared to be multifaceted approaches that involved all stakeholders. Monitoring of the impact on total sales of antibiotics by means of an interrupted time series (ITS) analysis and analysis of pharmacies selling antibiotics OTC using mystery clients were the methodologically strongest designs used.

**Conclusions:**

The published literature describing activities to enforce prescription-only access to antibiotics in LMICs is sparse and offers limited guidance. Most likely to be effective are comprehensive multifaceted interventions targeting all stakeholders with regular reinforcement of messages. Policy evaluation should be planned as part of implementation to assess the impact and effectiveness of intervention strategies and to identify targets for further activities. Robust study designs such as ITS analyses and mystery client surveys should be used to monitor policy impact.

**Electronic supplementary material:**

The online version of this article (10.1186/s12913-019-4359-8) contains supplementary material, which is available to authorized users.

## Background

Antimicrobial resistance (AMR) is a rapidly growing threat for global health [1]. Higher rates of AMR are observed in countries with high consumption of antibiotics. The higher resistance rates in southern and eastern Europe compared to northern Europe, for example, likely relate to the higher consumption of antibiotics in these regions [2]. Inappropriate prescribing and self-medicating are factors contributing to inappropriate use of antibiotics and may promote the emergence of resistant bacteria [3]. Self-medication with antibiotics is associated with incorrect self-diagnosis, short duration of treatment and inappropriate choice of therapeutic class and dosage [4–7]. Non-prescription use of antibiotics may also result from poor guidance regarding their use and safety by the pharmacist [8].

In many countries over-the counter (OTC) sales of antibiotics is prohibited by law [9], although that does not mean that antibiotics are not sold OTC in those countries [10]. Law enforcement requires adequate resourcing with well-functioning and effective registration systems for medicines and medicine suppliers, sufficient inspection capacity and a legal system able to impose penalties for breaches of regulations and these are not in place in many countries. Also, strict prohibition of OTC sales of antibiotics could lead to worse access to medicines in rural areas and amongst the poorest populations since pharmacies and chemical shops are often their first line of care [11]. Many low- and middle-income countries (LMIC) are currently moving towards prescription-only access to antibiotics [12]. They announce and undertake a variety of activities to support enforcement of existing laws and regulations, including governmental inspections, media campaigns, and educational activities.

Assessing the impact of a policy intervention is of utmost importance to identify whether the intended effects have been achieved and/or whether unintended effects occur [13]. In many cases this assessment is not planned nor carried out. Additionally, policy makers or researchers may be faced with methodological challenges if they wish to assess the impact of interventions. For example, there may be a lack of baseline data for before-after assessments, insufficient data points for a robust analysis or poor quality of data. Measurement of the effectiveness of interventions to enforce prescription-only access to antibiotics provides a useful example to examine the strengths and weaknesses of different methodological approaches to assessing the effectiveness of policy interventions, in this case, to ban or limit OTC sales of antibiotics. This systematic literature review aims to identify studies conducted in LMICs to enforce existing legislation and regulations prohibiting the OTC sales of antibiotics, assess the interventions used, their impact and examine the methods chosen for impact measurement including their strengths and weaknesses.

## Methods

The Preferred Reporting Items for Systematic Reviews and Meta-Analyses (PRISMA) methodological review framework was used to ensure systematic data collection and analysis of literature [14].

### Search strategy

A systematic literature search for studies reporting interventions supporting law enforcement of prohibiting OTC sales of antibiotics in LMICs was conducted in August 2018, in both PubMed and Embase. A combination of both Medical Subject Headings (MeSH) and non-MeSH key term for OTC sales, antibiotics and legislation using Boolean operators, were used for Pubmed. The full search strategy is included as an online resource (see Additional file 1). Also, reference lists of retrieved articles were searched for relevant articles by means of the snowball technique. All titles and abstracts were exported into ProQuest RefWorks to identify and remove duplicates.

#### Inclusion and exclusion criteria

Eligible studies had to report on the impact of single or multiple interventions supporting enforcement of laws prohibiting OTC sales of antibiotics. In this paper, these interventions are referred to as ‘law enforcement activities’. Studies were excluded if they did not contain information about the impact of law enforcement activities, did not focus on LMICs, were published before 2000 or both the title and abstract were not available in English. LMIC status was defined as countries having a LMIC status during the policy intervention (source: Worldbank). Both ‘OTC status’ and ‘non-prescription sales’ refer to medicines sold directly to a consumer without a prescription. These definitions do not include selling of antibiotics scheduled for pharmacist-initiated use. The articles were not selected based on quality of the study or risk of bias. A formal risk of bias assessment (e.g. using the GRADE approach) is not provided. Strengths and weaknesses of the studies were discussed and taken into consideration in the interpretation of the results as part of the main study aim.

### Data analysis

Heterogeneity of study designs, settings and outcome measures precluded any formal pooling of quantitative results. Therefore, a narrative synthesis was conducted. Information about study location, year of intervention, law enforcement activities undertaken, data sources, outcome measures, size of study, and study results were extracted from the studies. In addition, we report the strengths and limitations of the different methodological approaches used.

## Results

**Fig. 1:**
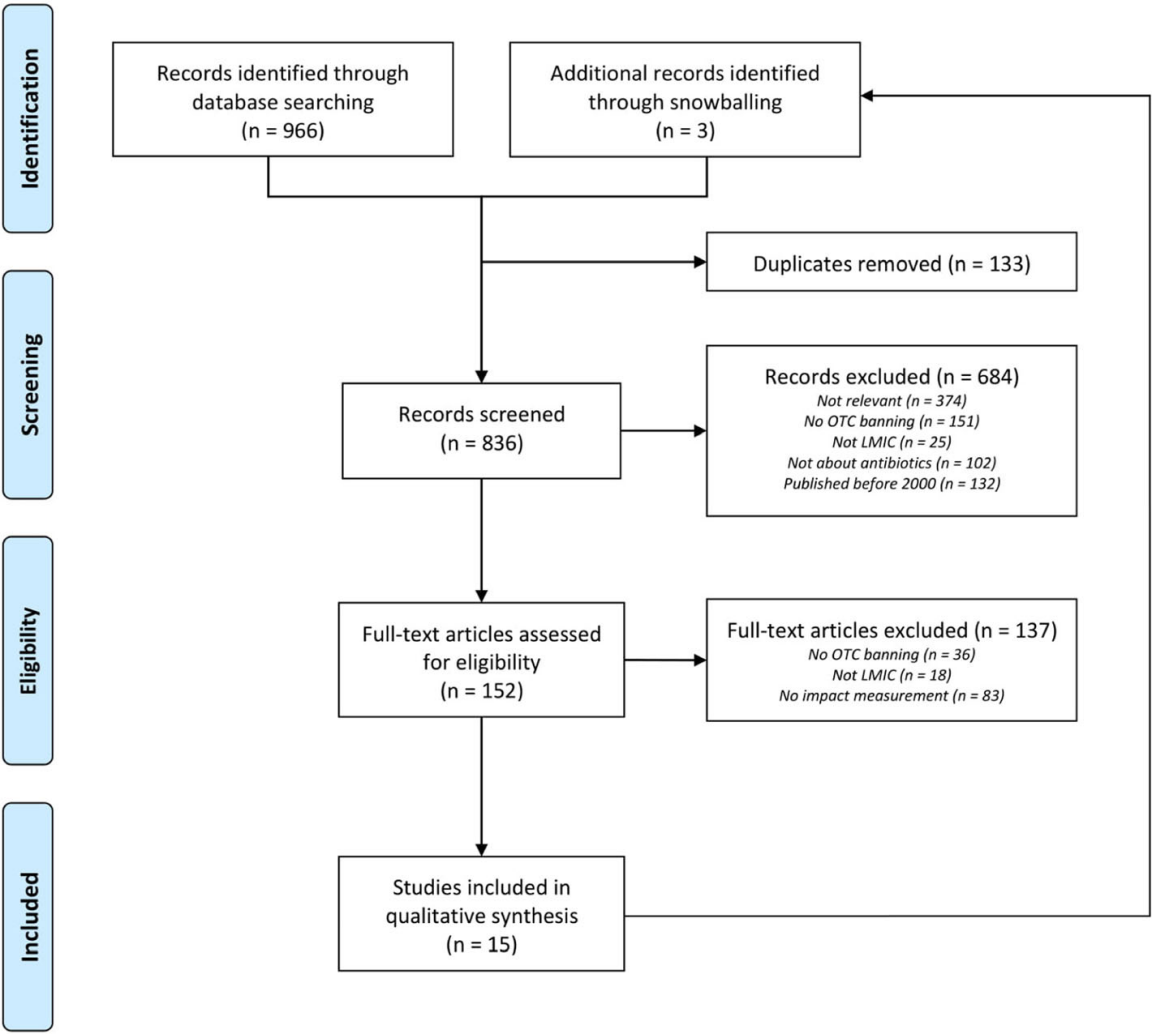
Preferred Reporting Items for Systematic Reviews and Meta-Analyses (PRISMA) flow diagram

The literature search resulted in 966 hits (335 hits in PubMed, 631 in Embase). Additionally, the snowballing method resulted in three additional hits. After excluding duplicates, 836 unique hits were screened for relevance based on titles. This resulted in 152 potentially relevant articles. After screening abstracts and/or full texts, 15 studies met the inclusion criteria (Fig. 1). In one case, only the abstract of the article was used because the full text was written in Spanish [15]. Several studies examined data from more than one country. The included studies reported on law enforcement in Brazil (*n* = 5) [16–20], Mexico (*n* = 2) [18, 19], Chile (*n* = 2) [15, 21], Colombia (*n* = 1) [21], Venezuela (*n* = 1) [21], Bosnia and Herzegovina (*n* = 2) [22, 23], Azerbaijan (*n* = 1) [24], North Macedonia (*n* = 1) [25], Vietnam (*n* = 3) [5, 26, 27] and Thailand (*n* = 2) [27, 28]. Countries were grouped by WHO region to account for potential regional and cultural differences in antibiotic use and implementation of regulations. The main characteristics of the studies including law enforcement activities, the method(s) of impact measurement and the key results are displayed in Table 1.

**Table 1:** Studies analysing the impact of law enforcement activities to prohibit OTC sales of antibiotics in lower- or middle-income countries

Study and setting	Outcome (data source)	Method	Intervention	Results	Methodological issues
Mattos et al. [16] 2017 Brazil	Changes in antibiotic sales (IMS Health data) AMR trend (*E. coli* resistance patterns)	Observational	• Retention of prescriptions • Inspections	Decrease in total sales of antibiotic not paralleled by decrease in AMR. Increasing trend in E. coli resistance seen in all antibiotic classes, except nitrofurans and folate pathway inhibitors.	Resistance data was obtained from one teaching hospital in Brazil and sales data from drugstores in the same metropolitan were analysed. Statistical analyses were used to verify linear resistance rates, but not to assess correlation between resistance patterns and differences in sales data. Other research indicated that low antibiotic sales do not necessarily correlate with low antibiotic resistance rates in LMICs [29].
Moura et al. [17] 2015 Brazil	Changes in total antibiotic use and shifts in therapeutic classes (IMS Health data)	ITS	• Retention of prescriptions • Inspections	• Direct effect on sales: − 1.87 DID • Penicillin, sulfonamide, macrolide sales decreased • The increasing trend in antibiotic sales tempered after the intervention (total increase in sales 2008–2012: 6.48 to 7.52 DID)	The study by Moura et al. included public antibiotic sales data to assess differences in direct policy impactbetween the private and public sector. Monthly antibiotic sales between January 2008 and December 2012 were analysed.
Santa-Ana-Tellez et al. [18] 2013 Brazil Mexico	Changes in total antibiotic use and shifts in therapeutic classes (IMS Health data)	ITS	• Retention of prescriptions (Brazil and Mexico) • Inspections (Brazil and Mexico) • Media campaign (Mexico)	Brazil • Direct effect on sales: −1.35 DID • Penicillin, sulfonamide, macrolide sales decreased • No change in increasing sales trend (total increase in sales Q1 2007-Q2 2012: 5.7 to 8.5 DID, + 49.3%) Mexico • Direct effect on sales: − 1.17 DID • Penicillin and sulfonamide sales decreased • No change in decreasing sales trend (total decrease in sales Q1 2007-Q2 2012: 10.5 to 7.5 DID, − 29.2%)	Sales data of antihypertensives were used as reference group to account for changes in medicine use independently of the OTC restrictions directed at antibiotics. Quarterly antibiotic sales between January 2007 and June 2012 were analysed.
Santa-Ana-Tellez et al. [19] 2015 Brazil Mexico	Changes in seasonal variation in penicillin sales (IMS Health data)	ITS	• Retention of prescriptions (Brazil and Mexico) • Inspections (Brazil and Mexico) • Media campaign (Mexico)	No significant differences in Brazil. Variation between penicillin sales in summer and winter decreased by 0.4 DID (− 36%) in Mexico.	High seasonal variation in penicillin use is suggested to be associated with more inappropriate use of antibiotics [30]. The authors suggest that appropriate use of antibiotics might mean that less antibiotics are sold without a prescription.
Lopes-Júnior et al. [20] 2015 Brazil	Changes in antibiotic sales in 3000 pharmacies	Before-after measurement	• Retention of prescriptions • Inspections	Tetracyclines (− 30.47%), sulfonamides (− 28.54%), macrolides (− 24.99%), and penicillins (− 20.46%)sales decreased. Only the sales of amoxicillin clavulanic acid seemed to increase (+ 9.14; not significant).	The study reported the annual sales data before and after the intervention. The results could not be seen in perspective of antibiotic sales patterns in other years around the intervention.
Wirtz et al. [21] 2013 Chile Colombia Venezuela	Changes in total antibiotic use and shifts in therapeutic classes (IMS Health data)	ITS	• Retention of prescriptions (Chile, Venezuela) • Inspections (Chile, Venezuela) • Media campaign (Chile, Colombia) • Involvement of pharmacists in designing enforcement (Chile)	Chile • Direct effect on sales: − 5.56 DID • Penicillin and sulfonamide sales decreased • No significant change sales trend (total decrease in sales Q1 1997-Q2 2002: 12.3 to 8.5 DID) Colombia • Direct effect on sales: on sales − 1.00 DID • Penicillin and sulfonamide sales decreased • No change in sales trend (total decrease in sales Q2 2002-Q4 2007: 9.16 to 6.76 DID) Venezuela • No direct reduction of antibiotic sales • Penicillin sales increased • No change in increasing sales trend (total increase in sales Q1 2003-Q4 2008: 9.99 DID to 15.38 DID)	Multiple countries were included in this study and analysed by means of the same method. Therefore, the policy impact in different could be compared to some extent. Since the researcher used an ITS analysis, they were able to directly relate the results to the policy intervention(s).
Bavestrello et al. [15] 2011 Chile	Changes in total antibiotic use (IMS Health data)	Observational	• Retention of prescriptions • Inspections • Media campaign • Involvement of pharmacists in designing enforcement	From 2000 to 2002 the impact of regulation persisted, but from 2003 to 2008 the total level of antibiotic use in Chile raised from 7.57 to 9.91 DID.	The authors did not take changes in antibiotic sales trend before the intervention into consideration. Therefore, it is almost impossible to make a strong conclusion based on this data.
Marković-Peković et al. [22] 2017 Bosnia and Herzegovina	Changes in OTC supply of antibiotics in 131 (2010) and 383 (2015) pharmacies	Mystery client method Before-after measurement	• Retention of prescriptions • Inspections • Media campaign	58% of investigated pharmacies sold antibiotics OTC to mystery clients presenting with self-diagnosed urinary tract infection in 2010 versus 18.5% in 2015.	The researchers assessed the policy impact on actual practice of pharmacists in supplying antibiotics OTC. The mystery clients presented at the pharmacies with the same symptoms during all visits to assure comparability.
Bojanić et al. [23] 2018 Bosnia and Herzegovina	Changes in total antibiotic use and shifts in therapeutic classes (National PHI)	Before-after measurement	• Retention of prescriptions • Inspections • Media campaign	The total sales of antibiotics did not significantly differ between 2010 and 2015 (17.6 and 16.8 DID respectively). Sales of amoxicillin-clavulanic acid and azithromycin increased	Since multiple law enforcement initiatives were instigated over the complete period of analysis, the researchers were unable to conduct an ITS analysis. Therefore, it is difficult to relate the results and assess the actual impact of the multifaceted intervention.
Abilova et al. [24] 2017 Azerbaijan	Changes in total antibiotic use and shifts in therapeutic classes (wholesaler import data)	Observational	• Retention of prescriptions • Inspections • Media campaign • Education of pharmacists	Total antibiotic use decreased by −9.08 DID between 2011 and 2015. Sales of tetracyclines, macrolides, and fluoroquinolones rose, while sales of beta-lactam antibiotics decreased.	Since multiple law enforcement initiatives were instigated over the complete period of analysis, the researchers were unable to conduct an ITS analysis. Therefore, it is difficult to relate the results and assess the actual impact of the multifaceted intervention.
Ivanovska et al. [25] 2018 North Macedonia	Changes in reported practice and knowledge regarding antibiotic use in 1203 clients	Survey Before-after measurement	• Retention of prescriptions • Inspections • Media campaign	Children self-medication rates decreased in 2015 and increased in 2016. No other changes were seen in client knowledge, attitude or behaviour.	Due to the short period between the survey rounds, the impact of the specific national media campaign on cliental knowledge could be assessed. Since the survey was conducted three times, some assumptions could be drawn about long-term impact. However, this methodology outcome does not capture the effect of interventions other than media campaigns.
Chuc et al. [26] 2002	Changes in OTC supply of antibiotics in 68 pharmacies	Mystery client method Quasi-experimental	• Inspections • Education of pharmacists	Fewer pharmacies dispensed cefalexin without a prescription after the interventions (from 95 to 56%) in the intervention pharmacies compared to control pharmacies (from 94 to 89%).	Due to the quasi-experimental design, the researchers could correct for other factors influencing the availability of antibiotics without a prescription, meaning that the results could be attributed to the policy intervention. However, due to the small sample size and since all pharmacies were located in one geographical area, the generalisability of the study can be questioned.
Chalker et al. [27] 2005 Vietnam Thailand	Changes in OTC supply of antibiotics in 68 (Vietnam) and 78 (Thailand) pharmacies	Mystery client method Quasi-experimental	• Inspections • Education of pharmacists	69% instead of 90% of pharmacies sold low dose antibiotics OTC after the intervention in Vietnam. No reduction in pharmacies selling antibiotics OTC in Thailand.	The results can be linked to the research interventions, due to the quasi-experimental study design that was used. However, due to the small sample size and since all pharmacies were located in one geographical area, the generalisability of the study can be questioned.
Arparsrithongsagul et al. [28] 2015 Thailand	Changes in OTC supply of antibiotics and Changes in reported practice and knowledge regarding antibiotic use in 116 groceries	Mystery client method and survey Quasi-experimental	• Inspections • Education of pharmacists	Fewer groceries sold antibiotics after the intervention (22.9% versus 79.2%), while there was little change in the control group.	The reduction in availability of antibiotics can be attributed to the intervention, due to the quasi-experimental study design that was used. All included grocery stores were located in one province. Therefore, the representativeness for Thailand as a whole can be questioned.
Chalker et al. [31] 2001 Vietnam	Changes in reported practice and knowledge regarding antibiotic use in 44 pharmacies	Survey Quasi-experimental	• Inspections • Education of pharmacists	Fewer pharmacists claimed they would sell cefalexin without a prescription after the intervention (20% versus 57%).	The results can be linked to the research intervention, due to the quasi-experimental study design that was used. Using reported practice by pharmacists makes the study vulnerable for social desirability bias.

*AMR* antimicrobial resistance, DID = defined daily doses per 1,000 inhabitants per day, *ITS* interrupted time series, *NA* not applicable, *OTC* over-the-counter, *PHI* Public health Institute

All studies focused on the prohibition of OTC antibiotics; in the Venezuelan case [21], the prohibition only applied to selected classes of antibiotics. The most common interventions were media campaigns (seven countries), government inspections (eight countries) and retention of prescriptions (eight countries). Education of pharmacists formed part of the suite of interventions in three countries. Only in Chile and Thailand, pharmacists or grocery store owners were involved in designing the law enforcement activities (Table 2).

**Table 2:**
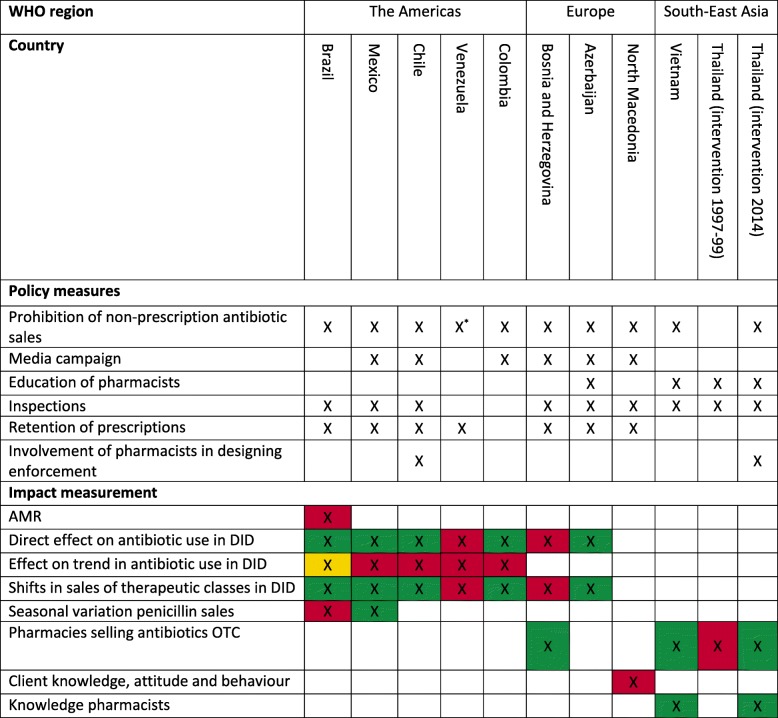
All policy measures taken by different countries to enforce the law prohibiting sales of antibiotics and the results of studies investigating the effect(s) of the policy measures

The colors of the boxes relate to the outcome of the study; green: significant positive effect, red: no effect, yellow: contradicting evidence

*AMR* Antimicrobial Resistance, DID = Defined daily doses per 1,000 Inhabitants Days, *OTC* Over-The-Counter

*Only part of the systematic antibiotics is prohibited to be bought without a prescription

Five main outcomes were reported in the 15 eligible studies: changes in AMR trends, changes in volumes of antibiotic use, change in patterns of antibiotic use, changes in level of OTC supply of antibiotics and changes in reported practice and knowledge about prescription-only access to antibiotics. One study used AMR data. Eight used antibiotic sales data by means of an interrupted-time-series (ITS) analysis or an observational study design. An ITS analysis is a quasi-experimental design, which applies statistical techniques such as segmented regression analysis to estimate both the direct policy impact on sales data (change in levels of consumption) as well as the impact on trends of consumption of antibiotics over time (e.g. does the slope differ from what was expected in the absence of the intervention). ITS studies can be conducted with or without a control group, the latter capturing changes in the prescribing environment apart from the intervention. Studies using an observational design, analysed annual antibiotic sales data by means of a trend analysis to see whether total antibiotic sales increased, decreased, or did not change during the period of analysis. It differs from the trend analysis in ITS studies in that ITS studies compare trends before and after the intervention, whilst these observational studies assess only one trend starting at the point of intervention. Two studies used surveys to assess the impact of an intervention on knowledge about antibiotic use and self-reported practice regarding antibiotics. The mystery client method was used in three studies; either by using before-and-after measurement or in a quasi-experimental controlled trial setting in which intervention groups were compared with non-intervention groups. One study used both the mystery client method and questionnaires.

### Main study results

All obtained information is grouped by country and WHO region. First, a short description of all reported policy measures is provided, followed by a description of the impact of the law enforcement as reported in the literature. A summary of all included studies, the methods used, and interpretation of the results is given in Table 1. Table 2 provides an overview of all law enforcement activities undertaken, and the reported impact is provided for each country.

#### The Americas

The Brazilian Health Regulatory Agency (ANVISA) implemented a policy enforcing the prohibition of OTC sales of all systemic antibiotics in 2010. They announced regular inspections of pharmacies to check the pharmacy’s compliance with the new policy. ANVISA did not carry out any information campaigns to inform patients about the policy change or to reduce inappropriate use of antibiotics. The effect of the enforcement in 2010 was measured in five different studies, which all reported a decrease in total antibiotic sales, based on both private commercial sales data from IMS Health and sales data reported by 3000 pharmacies in the private sector [16–20]. A decrease in sales of penicillins, sulphonamides, and macrolides [17, 18, 20] was reported, while the sales of fluoroquinolones seemed to increase [20]. The effect on antibiotic sales trend was assessed by means of an ITS analysis, however, the results differed between the two studies. Santa-Ana-Tellez et al. found no difference in antibiotic sales trend before and after the policy change [18], whereas Moura et al. reported a significant decrease in trend [17]. Moura et al. used quarterly antibiotic sales between January 2007 and June 2012, while Santa-Ana-Tellez et al. used monthly sales data between January 2008 and December 2012. The exact cause of this discrepancy in results is not clear. In addition, Santa-Ana-Tellez et al. (2015) investigated the impact of the policy intervention on appropriate antibiotic use by using seasonal variation of penicillin sales between summer and winter, and no difference was found [19]. Mattos et al. assessed the effect of the restriction of OTC sales of antibiotics on bacterial resistance trends in Brazil [16]. Annual resistance data of urinary *Escherichia coli* cultures from outpatients with a suspected urinary tract infection between 2009 and 2015 were compared with annual sales data of antibiotics (2008–2012) to study the impact of the policy intervention on bacterial resistance. Although an immediate decrease in antibiotic sales was seen, it did not appear to have any influence on the increasing bacterial resistance trend [16].

Law enforcement activities to reduce non-prescription sales of antibiotics started in 2010 and focused on both pharmacists and the general public in Mexico. Pharmacies had to retain and register all prescription data for systemic antibiotics. The ministry of health (MoH) announced significant penalties and revocation of licenses in case of non-compliance [32]. In addition, patients were informed about the policy change by means of a media campaign [33]. Santa-Ana-Tellez et al. reported a direct reduction in antibiotic sales in the private sector (assessed using IMS Health data) after the intervention [18]. Sales of penicillins and sulphonamides decreased significantly [18]. Moreover, the seasonal variation decreased significantly from 1.1 defined daily dose per thousand inhabitants per day (DID) to 0.7 DID [19]. While there was a change in the level of consumption, there was no change was seen in trends of antibiotic sales over time before and after the intervention [18].

Chile moved towards prescription-only access to antibiotics in 1999. The MoH targeted both pharmacies and the general public in enforcing the regulation. In addition, pharmacists were involved in the design of the enforcement. Antibiotics were removed from a list of medicines having sales incentives provided for pharmacy staff. Wirtz et al. assessed the impact on changes in antibiotic use and changes in patterns of antibiotic use in three different countries by means of an ITS analysis. They reported a considerable decrease in overall private antibiotic sales (− 5.56 DID; baseline: 14.5 DID) after the intervention [21]. Moreover, the trend in antibiotic sales over time seemed to reduce in the 3 years after the implementation compared to before the intervention [21]. However, there was no evidence of persisting long-term effects of the intervention. After 2002 the sales increased again, resulting in similar numbers of antibiotic sales in 2008 compared to before the intervention [15].

In 2005, Colombia only focussed on the district of Bogota for reducing OTC sales of antibiotics. An information campaign targeted the general public both before and during the intervention. However, no information about retaining prescriptions and inspections in pharmacies was reported [21]. A small decrease in private antibiotic sales (− 1.00 DID; baseline 8 DID) was observed after the intervention by means of an ITS analysis. The total sales of antibiotics in the private sector decreased over time, although no change in trend of antibiotic sales was seen that could be attributed to the intervention [21].

OTC sales of quinolones, macrolides-lincosamides, third-generation cephalosporins and rifampicin were prohibited from 2006 onwards in Venezuela. Law enforcement only targeted pharmacist by obliging them to retain prescriptions. However, no records of actual inspections were found. The new regulation seemed to have no effect on reducing antibiotic sales, while penicillin use sharply increased in Venezuela [21].

In summary, all studies assessing policy impact in The Americas used antibiotic sales data. In Brazil, a direct reduction in antibiotic sales in the private sector was reported after the policy intervention. There was inconsistency in evidence about differences in trends of consumption of antibiotics before and after the intervention in Brazil. No impact was found on seasonal variation Brazil, whereas the seasonal variation decreased in Mexico. In both Mexico and Colombia, the antibiotic sales decreased after the intervention, but no impact on sales trends was found. The largest direct impact on antibiotic sales was seen in Chile, whereas no impact was reported in Venezuela.

#### Europe

A multifaceted program targeting patients, pharmacists and other healthcare workers was implemented by the regulatory authorities in the Republika Srpska (Bosnia and Herzegovina) from 2010 onwards. Media campaigns were initiated, and a guideline was developed for pharmacy workers to assist them in decisions regarding providing legal OTC medicines or referral of the patient to a doctor. The impact on OTC supply of antibiotics was assessed by means of the mystery client method. The number of pharmacies selling antibiotics significantly decreased between 2010 and 2015 (from 58 to 18.5%) [22]. However, no effect was seen on total sales of antibiotics reported by the Bosnia and Herzegovina Public Health Institute between these years; the total antibiotic sales remained stable, which might suggest that more patients visited doctors to obtain a prescription [23].

Azerbaijan also implemented a multifaceted program targeting pharmacists and the general public from 2011 onwards to reduce OTC sales of antibiotics. Throughout the period of analysis, media campaigns were set-up every year in April and November intended to educate pharmacists and the general public about appropriate antibiotic use. In addition, pharmacists were educated about the changed regulation during inspections by governmental workers. A substantial decrease in total sales of antibiotics (based on import records) was reported between 2011 and 2015 (from 17.1 DID to 8.02 DID).

The policy intervention in North Macedonia took place in 2014 and 2015 and targeted both health care workers and the general public. Ivanovska et al. assessed change in patient knowledge and self-reported practices regarding non-prescription use of antibiotics by the parents and their children [25]. A survey of the general public was conducted three times; before the interventions (2014, *n* = 403), after a national media campaign about antibiotic use (2015, *n* = 400) and after implementing fines for pharmacies selling antibiotics without a prescription (2016, *n* = 400). This study showed an initial impact of the media campaign by an increasing percentage of children using antibiotics with a prescription (89 to 95%). However, this impact subsided during the third round of questionnaires after 1 year. No effect was seen in self-medication practices in the parents [25].

#### South-East Asia

In Vietnam, 29 pharmacies were exposed to a regulatory enforcement, educational, and peer influence intervention between 1998 and 1999 aimed to reduce the number of pharmacists selling antibiotics without prescription. The interventions led to a significant drop in pharmacies selling cefalexin without a prescription to a mystery client (56%) compared to reference pharmacies that were not exposed to the intervention (89%) [26]. In addition, Chalker et al. assessed the impact of the interventions on both knowledge and reported change in practice by means of a survey amongst pharmacies. The number of pharmacists indicating that they would sell cefalexin without a prescription in a questionnaire also decreased in the intervention group (20% instead of 57%) [31].

The same interventions were conducted in 39 pharmacies in Thailand between 1997 and 1999. However, no significant differences in number of pharmacies selling cefalexin without a prescription before and after the interventions were reported [27]. A second intervention that took place in Thailand around 2014 targeted grocery store owners in 20 different villages. Community leaders and government officers visited grocery owners to teach them about responsible antibiotic use and regulations regarding OTC sales of antibiotics [28]. A study by Arparsrithongsagul et al. assessed the impact of the intervention by means of a quasi-experimental design for the mystery client- and survey method. The rate of grocery stores having antibiotics in stock (penicillin in particular) decreased significantly in the villages that were exposed to the intervention (79% vs 23%) compared to the unexposed pharmacies (88% vs. 85%). These results were strengthened by an increase in knowledge of grocery store owners about antibiotic use, especially on regulation of antibiotic sales [28].

### Methodological considerations

The studies illustrate a number of methodologies and outcome measures of varying robustness and utility for evaluating the impact of interventions and informing policy actions. The mystery client method closest approaches the measurement of actual practices of pharmacists. In a study conducted in Vietnam [31], only 20% of pharmacists stated that they would sell cefalexin without a prescription post-intervention, yet 56% of the same sample of pharmacies still sold cefalexin to mystery clients without a prescription [26]. This emphasises the importance of measuring actual behaviour instead of self-reported behaviour. However, mystery client studies are resource intensive undertakings. There were four examples in this review of repeat studies using mystery clients to assess changes in practices over time [22, 26–28]. Researchers need to be careful in both selection of time points to account for seasonal variations and clinical status of the patient to prevent erroneous conclusions.

Most studies relied on the use of sales data to assess the impact of interventions on volumes or patterns of consumption of antibiotics. To account for changes in medicine use independently of the OTC restrictions, some researchers used sales data of antihypertensives or antibiotic sales data in the public sector as a reference group assuming that the interventions did not affect use in these cases because antibiotics are only accessible by prescription in the public sector [17, 18]. Sales data have the advantage of being readily available at reasonable cost, facilitating tracking of changes and patterns over time. However, changes in antibiotic sales data will reflect the impact of all types of interventions rather than specific elements. Any changes in patients’ knowledge and practice regarding antibiotic usage would likely be attributable to the impact of media campaigns but would not be identified separately in changes in overall volumes of use. This argues for a suite of assessment tools to enable assessment of the impact of the different strategies applied.

Changes in sales of specific antibiotic classes or seasonal variation in penicillin sales will facilitate the evaluation of more targeted interventions to change practice. An important corollary is the need to consider unintended consequences. Shifts away from supply of some classes of antibiotics may be offset by changes to other classes, such as quinolones or third- and fourth-generation cephalosporins that may also be undesirable. System impacts should also be monitored.

Where a policy intervention is implemented at a point in time, an ITS analysis can be conducted [34]. This analysis makes it possible to attribute the observed effect to the intervention and makes this method more robust compared to an observational study design using annual antibiotic sales data. In the studies included in this literature review, only the law enforcement activities in the South American countries could be defined as such. The multifaceted interventions in Azerbaijan and Bosnia and Herzegovina were conducted throughout the whole period of analysis, which made it impossible to define one moment in time. Therefore, time trends in antibiotic use were conducted in the studies assessing the impact of the interventions in these countries. However, many other factors can contribute to changes in antibiotic usage and therefore conclusions on the effectiveness of the interventions need to be more confined.

Multiple studies used a quasi-experimental controlled design in assessing impact on changes in OTC supply of antibiotics or self-reported practice and knowledge. These studies compared pharmacies that were exposed to interventions with unexposed pharmacies to correct for other variables that can affect the outcome. However, these studies [26–28, 31] usually include small sample sizes which is unfavourable for the generalisability of the study. Studies using before-and-after measurements to assess the impact on OTC supply of antibiotics included larger samples compared to the ones using a quasi-experimental study design, making the sample more representative for the whole country.

## Discussion

This systematic literature review shows that research on the impact of law enforcement activities to reduce OTC access to antibiotics in low and middle-income countries is sparse. We found 15 eligible studies that reported the impact of interventions undertaken in 10 LMICs. Most law enforcement activities included regulatory (governmental inspections), managerial (involvement of pharmacists in designing interventions and retention of prescriptions) and educational (media campaigns and education of pharmacists) interventions. The impact of law enforcement activities varied greatly between countries. This can be explained in part by differences in policy measures and strictness of enforcement, differences in socio-economic factors, or differences in overall medication use. Table 2 shows that countries where no impact of interventions was shown were the countries which implemented least interventions (e.g. Venezuela and Thailand (intervention 1997–1999)). This points towards the need of implementing comprehensive multifaceted interventions. In addition, the study from North-Macedonia clearly showed that the effect ceased after the campaign had stopped. A wide range of methods and outcomes measures to quantify the effect of the enforcement was found. This makes it difficult to quantify the differences in actual impact of law enforcement between studies, countries and therefore between measures that were undertaken to enforce prohibition of OTC sales of antibiotics. It is for example very difficult to compare data on antibiotic use with a decrease in the number of pharmacies selling antibiotics without a prescription. The Bosnia and Herzegovina case indicated that a decrease in pharmacies selling antibiotics not necessarily leads to a decrease in total sales of antibiotics or specific antibiotic classes in a country [22, 23]. Whether the policy interventions can still be considered effective in this country depends on which outcome measure is regarded most important. Using multiple outcomes is essential in assessing policy impact. When looking only at OTC sales of medicine dispensers, for example, one might miss other system impacts such as doctors writing prescriptions or patients using antibiotics more appropriately. Therefore, the policy conclusions and recommendations from the studies (see Table 3) are based on consistency of effect in a positive direction.

**Table 3:** Policy recommendations for a multifaceted intervention to enforce laws prohibiting non-prescription sales of antibiotics

Policy recommendation	
A comprehensive multifaceted intervention is needed to enforce laws prohibiting non-prescription sales of antibiotics. Monitoring of the impact on total sales of antibiotics and pharmacies selling antibiotics OTC is strongly advised. Based on current literature it is recommended to: • Involve pharmacists in designing the law enforcement activities; • Educate medicine dispensers about the regulation and risks of irresponsible use of antibiotics; • Implement media campaigns focussing on educating patients about the risks of self-medication with antibiotics on a regular basis over a longer period of time; • Conduct regular inspections for retention of prescriptions in pharmacies by governmental workers during which penalties do not necessarily have to be implied in case of non-compliance.	

*OTC* over-the-counter

In most countries, law enforcement related to the non-prescription sale of all systemic antibiotics. Restriction of OTC sales of only a selected group of systemic antibiotics (reserve antibiotics and tuberculosis medicines) led to an increase in sales of penicillins in Venezuela [21]. A similar effect might be expected in India, since the recent prohibition of OTC sales of a selected group of antibiotics [35]. Since broad-spectrum penicillins and other broad-spectrum antibiotics are the most frequently sold antibiotic classes without a prescription [3, 36], it is important to include these classes to accomplish a significant reduction of antibiotic sales. In LMICs, however, children are still more likely to die due to a lack of access to effective and affordable antibiotics compared to antibiotic resistance [37]. Therefore, a balance has to be struck between reducing excess use of antibiotics and improving access to needed antibiotics in moving towards prescription-only access to antibiotics. This balance cannot be accomplished by only prohibiting OTC sales. There is a need for public information campaigns on more appropriate use of antibiotics [38] and improvement of prescription practices by doctors to reduce unnecessary antibiotic usage [39].

Both media campaigns and legislation requiring retention of antibiotic prescriptions seemed to have impact only when undertaken as longer-term interventions. In Azerbaijan, the public campaign was conducted twice yearly for multiple years [24]. This might have contributed to the significant, persisting decrease in antibiotic sales between 2011 and 2015. A repeating commercial or advertisement makes the population familiar with the content and is more likely to have impact [38, 40]. In contrast, the media campaign in Chile was comprehensive but only operated before and during the implementation of the policy change. This might have influenced the increase in antibiotic sales that was seen 3 years after the law enforcement started [15]. To make a difference, public campaigns should focus on changing the public attitude and behaviour regarding antibiotics [38]. Moreover, public health campaigns in other disciplines have shown that repeated exposure to the campaign often results in a sustained impact.

Only studies conducted in Azerbaijan, Vietnam and Thailand included education of pharmacists, and most of them were considered to be successful. In Azerbaijan, pharmacists were educated about the prescription-only status of antibiotics when they were visited by the governmental inspectors [24]. It is time-consuming to educate all pharmacists face-to-face. However, pharmacies needed to be visited by a governmental inspector anyway to be checked for compliance. Taking the opportunity to educate pharmacists at the same time is an interesting intervention that seemed to contribute to a reduction in total antibiotic sales. In Thailand, influential village people were trained to educate medical sellers in their village about responsible antibiotic use and regulatory measures [28]. This could be a very effective way to reach all untrained medical sellers in rural areas. Retention of prescriptions was mandatory in many included cases. The thoroughness of the inspections of the retained antibiotic prescriptions was unfortunately poorly described in literature. Unsuccessful law enforcement initiatives, like the Venezuela case, failed to impose sanctions for non-compliance [21]. However, in Vietnam and Azerbaijan, the government conducted visits by regulatory inspectors, but no fines were to be given. Despite the lack of sanctions, the law enforcement was effective in both countries [24, 26]. There is insufficient evidence to draw a conclusion about the impact of penalties on behavior of pharmacies in this case.

Ultimately, the policy changes and enforcement of prescription-only access would be expected to encourage more rational use of antibiotics and positively affect antimicrobial resistance patterns in a country. A potential explanation for the lack of impact on AMR patterns in Brazil is that the policy measures did have a direct effect on the level of use as confirmed in some of the other studies in this review [17, 18], but could not change the underlying trend of an overall increase in antibiotic sales. A recent study about the influence of anthropological and socioeconomic factors on global AMR concluded that reduction of antibiotic consumption will not be sufficient to control AMR [29]. In addition to improving antibiotic dispensing practices, countries should focus on improving sanitation, increasing access to clean water, and increasing public health-care expenditure [29]. AMR patterns therefore do not seem the most appropriate outcome measure to assess the impact of interventions aiming at prohibiting OTC sales of antibiotics.

Most studies included in this review assessed policy impact based on antibiotic sales data from the private sector. This sector is suggested to be most affected by the law enforcement activities, since patients already need a prescription when getting medicines dispensed in the public sector. Moura et al. confirmed the absence of an impact of law enforcement in the public sector [17]. The ITS analysis is the preferred statistical method to assess policy impact by means of total antibiotic sales, since it is a strong quasi-experimental approach for evaluating direct changes in levels of consumption as well as the long-term effect and relates the impact to the policy intervention [34]. Moreover, it covers impact on both change in levels of consumption as well as the impact on trends of consumption of antibiotics over time due. However, many factors can influence the total amount of antibiotic sales. For example, accessibility, availability, affordability, reimbursement and other policy changes involving antibiotics can affect the total use of certain medicines [41].

Antibiotic sales data can also be used as a proxy measure for the quality of antibiotic use in a certain country by means of shifts in antibiotic sales and seasonal variation [30]. Moreover, sales data can be used to measure unintended effects of restricting OTC sales of antibiotics. Santa-Ana-Tellez et al. also identified increasing sales of non-steroidal anti-inflammatory drugs (NSAIDs) and cough and cold medications which coincided with decreasing antibiotics sales [42]. Since NSAID may have severe side effects such as stomach bleedings [43], this increase is potentially unintended. Few studies included in this review reported on unintended consequences. However, it is an important feature in designing the evaluation of policy interventions. In this case important consequences are shifts to other classes of antibiotics or other medicines [19, 23, 24], compensatory changes in the health system with greater burden on PHC for doctors to write prescriptions [44], employment of doctors by pharmacies [32, 45], the emergence of black markets [33], or limited access to antibiotic prescriptions in poor rural communities [46].

The mystery client method makes it possible to measure the impact on actual practice of pharmacists in selling antibiotics OTC [47]. However, the number of pharmacies selling antibiotics OTC highly depends on the patient that is asking for the antibiotics and the clinical condition. A study conducted in Spain revealed that patients visiting a pharmacy with symptoms corresponding to urinary tract infection were far more likely to receive antibiotics OTC compared to patients having complaints indicating acute bronchitis (81.1 vs 32.9%) [48]. Moreover, it can be a costly and time-consuming method, does not provide continuous information over time, and often acquires data from a small part of the pharmaceutical system of a country. However, interventions can be targeted at the pharmacy sector if OTC sales of antibiotics are common. Surveys to assess pharmacists’ self-reported practices regarding OTC sales of antibiotics are an inaccurate method to test the impact of law enforcement. It can be assumed that pharmacists tend to give socially desirable answers to the question if they would sell antibiotics OTC, because they know it is prohibited by law. The studies conducted in Vietnam confirmed this, since a large difference was found between self-reported practice and actual practice in the same study population [26, 31]. In addition, the study by Ivanovska et al. showed the importance of the period of time in which the impact is measured, since it might fade over time [25].

Most countries were classified as middle-income countries during the period of law enforcement, except Vietnam and Thailand in 1997 and 1999. It is clear that there is a paucity in data from lower income countries (LICs) and countries from the African and Middle Eastern WHO regions. Multiple studies have reported about the issue of OTC sales of antibiotics in these countries [10], but no initiatives to enforce laws regarding non-prescription sales of antibiotics in Africa have been described according to our review. A possible explanation for this is that LICs often lack regulatory resources, systems to track antibiotic sales, healthcare capacity and access to healthcare to strictly enforce laws and monitor the impact of their measures [49].

The period during which law enforcement took place varied greatly between countries over a period of around 20 years (1997–2016). In this period, methods for communication of policy changes, general knowledge of antibiotics and antibiotic treatment strategies have changed. This makes it difficult to generalize findings regarding the impact of communication strategies. This review only included articles written in English. Therefore, we were not able to include the full text of the paper by Bavestrello et al., but only the English abstract [15]. Another potential limitation of this review is that only two databases were searched, i.e. PubMed and Embase. Although the snowballing technique was used, we might have missed articles on this topic as well as grey literature. Many different study designs have been used to measure policy impact. Due to heterogeneity of study designs it was impossible to pool data from the different studies, which is a limitation of this study. However, the wide range of different included studies also reflects the broad literature search that has been conducted. The strength of this review is that it is the first to provide a full overview of all different methods that have been used to assess impact of law enforcement activities in moving towards prescription-only access to antibiotics.

## Conclusions

There are only a few published studies assessing the impact of law enforcement to reduce OTC sales of antibiotics in LMICs, and these offer limited guidance to other authorities who may wish to enforce existing legislation in their own settings. Multiple methods with different outcome measures have been used to assess impact of these interventions. These differences make it hard to compare outcomes and determine the effectiveness of specific law enforcement activities. However, involvement of pharmacists in the design of interventions, education of medicine dispensers, media campaigns targeted at the general public conducted on a regular basis over a period of time and inspections by governmental workers seemed most effective when implemented as part of a package of measures. ITS analysis and the mystery client method are preferred methods for evaluation of the impact of interventions because they allow for measuring long-term policy impact and actual practice of medicine dispensers, respectively. The appropriate methods should be chosen based on available resources and duration of law enforcement activities. Evaluation of policy impact should be planned and undertaken prior to implementing policy interventions to inform on policy development and guide targets for additional interventions.

## Additional file

Additional file 1 Search strategy. Full search strategy for PubMed. (DOCX 14 kb)

## Data Availability

Most of the studies included in the literature review are publicly available and can be obtained online. The other papers can be requested from the authors of the document. Aukje Mantel-Teeuwisse can be contacted for questions regarding the data we used.

## Abbreviations

AMR: Antimicrobial resistance
DID: Defined daily dose per thousand inhabitants per day
ITS: Interrupted-time-series
LMIC: Low- and middle-income countries
MoH: Ministry of health
NSAID: Non-steroidal anti-inflammatory drugs
OTC: Over-the-counter
PRISMA: Preferred Reporting Items for Systematic Reviews and Meta-Analyses
WHO: World Health Organization
