# Supplementary material for: Assessing the impact of law enforcement to reduce over-the-counter (OTC) sales of antibiotics in low- and middle-income countries; a systematic literature review
Source: BMC Health Serv Res. 2019 Jul 31;19:536. doi: 10.1186/s12913-019-4359-8 (PMC6670201; doi:10.1186/s12913-019-4359-8)
Supplement: Supplementary file 1 — Search strategy. Full search strategy for PubMed. (DOCX 14 kb) [file 12913_2019_4359_MOESM1_ESM.docx]

# **Search Strategy (Pubmed)**

(“non-prescription drugs”[MeSH] OR non-prescription drugs OR self-medication OR over-the-counter OR otc) AND (“anti-bacterial agents”[MeSH] OR anti-bacterial agents OR antibiotic use OR antibacterial OR antibiotic*) AND (legislation OR policy OR regulation OR enforcement OR prohibition)

## **MeSH terms**

All used MeSH terms included toe following entry terms and other MeSH terms:

- “Non-prescription drugs”
  - Entry terms: Drugs, Nonprescription; Medicines, Patent; OTC Drugs; Drugs, OTC; Over-the-Counter Drugs; Drugs, Over-the-Counter; Over the Counter Drugs; Patent Medicines; Drugs, Non-Prescription; Drugs, Non Prescription; Non-Prescription Drugs; Non Prescription Drugs
  - Other MeSH terms included: “Behind-the-Counter Drugs”
- “Anti-bacterial agents”
  - Entry terms: Agents, Anti-Bacterial; Anti Bacterial Agents; Antibacterial Agents; Agents, Antibacterial; Anti-Bacterial Compounds; Anti Bacterial Compounds; Compounds, Anti-Bacterial; Bacteriocidal Agents; Agents, Bacteriocidal; Bacteriocides; Anti-Mycobacterial Agents; Agents, Anti-Mycobacterial; Anti Mycobacterial Agents; Antimycobacterial Agents; Agents, Antimycobacterial; Antibiotics; Antibiotic
  - Other MeSH terms included: Antitreponemal Agents; Antitubercular Agents; Antibiotics, Antitubercular; beta-Lactamase Inhibitors; Leprostatic Agents
